# In Vitro Enzymatic Digestibility of Glutaraldehyde-Crosslinked Chitosan Nanoparticles in Lysozyme Solution and Their Applicability in Pulmonary Drug Delivery

**DOI:** 10.3390/molecules24071271

**Published:** 2019-04-01

**Authors:** Nazrul Islam, Hui Wang, Faheem Maqbool, Vito Ferro

**Affiliations:** 1 Pharmacy Discipline, School of Clinical Sciences, Faculty of Health, Queensland University of Technology (QUT), Brisbane, QLD 4000, Australia; h82.wang@connect.qut.edu.au; 2 Institute of Health and Biomedical Innovation, QUT, 60 Musk Avenue, Kelvin Grove, Brisbane, QLD 4059, Australia; 3 School of Pharmacy, The University of Queensland, Woolloongabba, QLD 4102, Brisbane, Australia; faheemthepharmacist@gmail.com; 4 School of Chemistry and Molecular Biosciences, The University of Queensland, Brisbane, QLD 4072, Australia; v.ferro@uq.edu.au; 5 Australian Infectious Diseases Research Centre, The University of Queensland, Brisbane, QLD 4072, Australia

**Keywords:** chitosan, glutaraldehyde, nanoparticles, lysozyme, degradation, pulmonary drug delivery

## Abstract

Herein, the degradation of low molecular weight chitosan (CS), with 92% degree of deacetylation (DD), and its nanoparticles (NP) has been investigated in 0.2 mg/mL lysozyme solution at 37 °C. The CS nanoparticles were prepared using glutaraldehyde crosslinking of chitosan in a water-in-oil emulsion system. The morphological characterization of CS particles was carried out using scanning electron microscopy (SEM) and Transmission Electron Microscopy (TEM) techniques. Using attenuated total reflectance Fourier transform infrared (ATR-FTIR) and UV-VIS spectroscopy, the structural integrity of CS and its NPs in lysozyme solution were monitored. The CS powder showed characteristic FTIR bands around 1150 cm^−1^ associated with the glycosidic bridges (C-O-C bonds) before and after lysozyme treatment for 10 weeks, which indicated no CS degradation. The glutaraldehyde crosslinked CS NPs showed very weak bands associated with the glycosidic bonds in lysozyme solution. Interestingly, the UV-VIS spectroscopic data showed some degradation of CS NPs in lysozyme solution. The results of this study indicate that CS with a high DD and its NPs crosslinked with glutaraldehyde were not degradable in lysozyme solution and thus unsuitable for pulmonary drug delivery. Further studies are warranted to understand the complete degradation of CS and its NPs to ensure their application in pulmonary drug delivery.

## 1. Introduction

Chitosan (CS) is a linear polysaccharide composed of repeating glucosamine (GlcN) and (some) N-acetylglucosamine (GlcNAc) units linked by β-(1→4) glycosidic bonds. It is an amphiphilic polymer obtained by deacetylation of naturally occurring chitin, which has been extensively studied for the delivery of various drugs, vaccines, genes and chemotherapeutic agents [1,2,3]. Using different crosslinkers (glutaraldehyde, tripolyphosphate, glutaric acid, glyceraldehyde, formaldehyde, and genipin), chitosan micro/nanoparticles are prepared by various techniques. CS nanoparticles (NPs) have been demonstrated to enhance mucoadhesiveness or cellular absorption upon lung delivery [4,5,6,7].

Recently, CS micro/nanoparticles have been investigated as carriers for pulmonary drug delivery [2,8,9,10,11,12]. Direct delivery of drugs into the lungs is of increasing interest owing to the thin epithelial layer and large surface area available for drug absorption that enables therapeutic benefits of drugs at very low doses. CS is recognized as non-toxic, biocompatible with pulmonary epithelial cells [9,13] and biodegradable [9,14,15], and has been widely investigated in lung drug delivery systems [6]. CS is also known as a dispersibility enhancer of particles leading to increased deposition into the lungs [9].

**Scheme 1:**
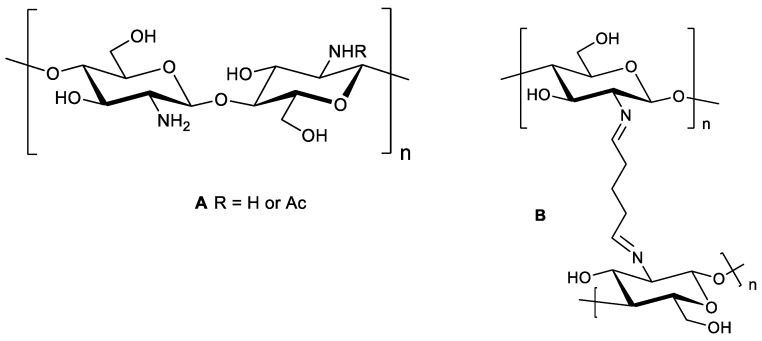
(**A**) Chitosan. (**B**) Chitosan chemically crosslinked with glutaraldehyde.

Using glutaraldehyde as a crosslinker, CS NPs with or without drug loading have been investigated for drug delivery and medical purposes [11,12,13,16,17,18,19]. Depending on the reaction conditions, i.e., the concentrations of glutaraldehyde and chitosan, and the pH of the reaction medium, the structures of the crosslinked products are varied. CS crosslinked with glutaraldehyde produces a covalently bonded chitosan-glutaraldehyde conjugate (Scheme 1) [20].

Glutaraldehyde is a known toxic chemical; however, no quantitative data are available regarding the absorption of inhaled glutaraldehyde and its subsequent toxicity [21]. Occupational exposure to glutaraldehyde has been reported to be associated with respiratory tract irritation. Zissu et al. [22] reported no histopathologic evidence of glutaraldehyde-induced lesions in the lungs of mice repeatedly exposed to glutaraldehyde vapor at 0.1 ppm for 78 weeks; whereas, Halatek et al. [23] reported lesions in the pulmonary epithelium (rat model) exposed to glutaraldehyde vapor at 0.1 ppm for 4 weeks. No studies of glutaraldehyde toxicity upon inhalation of glutaraldehyde crosslinked CS NPs have been reported. In our lab, we have found (unpublished data) that drug-loaded CS NPs did not produce any changes to lung epithelial cells after pulmonary administration in a mouse model after exposure for 24 h. Excess, unreacted glutaraldehyde used in the preparation of crosslinked CS NPs could potentially be released from the NPs and cause respiratory tract irritation if the NPs are not thoroughly washed. Therefore, if the crosslinking is done in such a way that there is no unreacted glutaraldehyde, the prepared NPs would not cause any lung irritation (except the effect of CS). More studies are warranted to understand the possible toxic effects of glutaraldehyde or CS-glutaraldehyde complexes in biological systems.

CS and its crosslinked NPs are regarded as biodegradable; however, the glutaraldehyde cross-linked CS particles formed via covalent bonds are thought to be very rigid and degradability in physiological fluids is not known. Glutaraldehyde crosslinked CS NPs have been investigated in a large number of drug delivery studies [11,12,17,18,19,24,25]. The degradation of various types of CS and their micro/nanoparticles prepared by other crosslinking techniques has been investigated in various studies [8,9,14,26,27]; however, the in vitro/in vivo degradation of CS and its particles intended for pulmonary drug delivery is not fully understood.

It is well known that the lysozyme enzyme secretions in the body are responsible for degrading CS or CS NPs [8,9]. Due to the complex structure of CS particles, it is unknown whether such structures are accessible to lysozyme in the lung fluids where the maximum concentration of lysozyme is 0.2 mg/mL [28]. Therefore, the present study focused on synthesizing CS NPs crosslinked with glutaraldehyde using the method demonstrated by Wang et al. [12] and to understand the in vitro degradation of the NPs in lysozyme solution in phosphate buffered saline (PBS, pH 7.4) at 37 °C. Most researchers investigated the CS degradation in different media by determining the molecular weight of CS, the viscosity of the experimental sample, particle size and weight remaining of samples after a particular time interval of incubation in lysozyme solution. These methods are unable to elucidate any chemical changes in the CS structure by lysozyme. In the present study, the experimental data from Fourier transform infrared spectroscopy with attenuated total reflectance (ATR-FTIR) and UV-Vis spectroscopy was evaluated to understand the degradation behavior and applicability of glutaraldehyde crosslinked CS NPs as a carrier for lung drug delivery.

## 2. Results and Discussion

### 2.1. Structural Integrity Studies of CS by ATR-FTIR Analysis

#### 2.1.1. CS Original Powder

**Figure 1:**
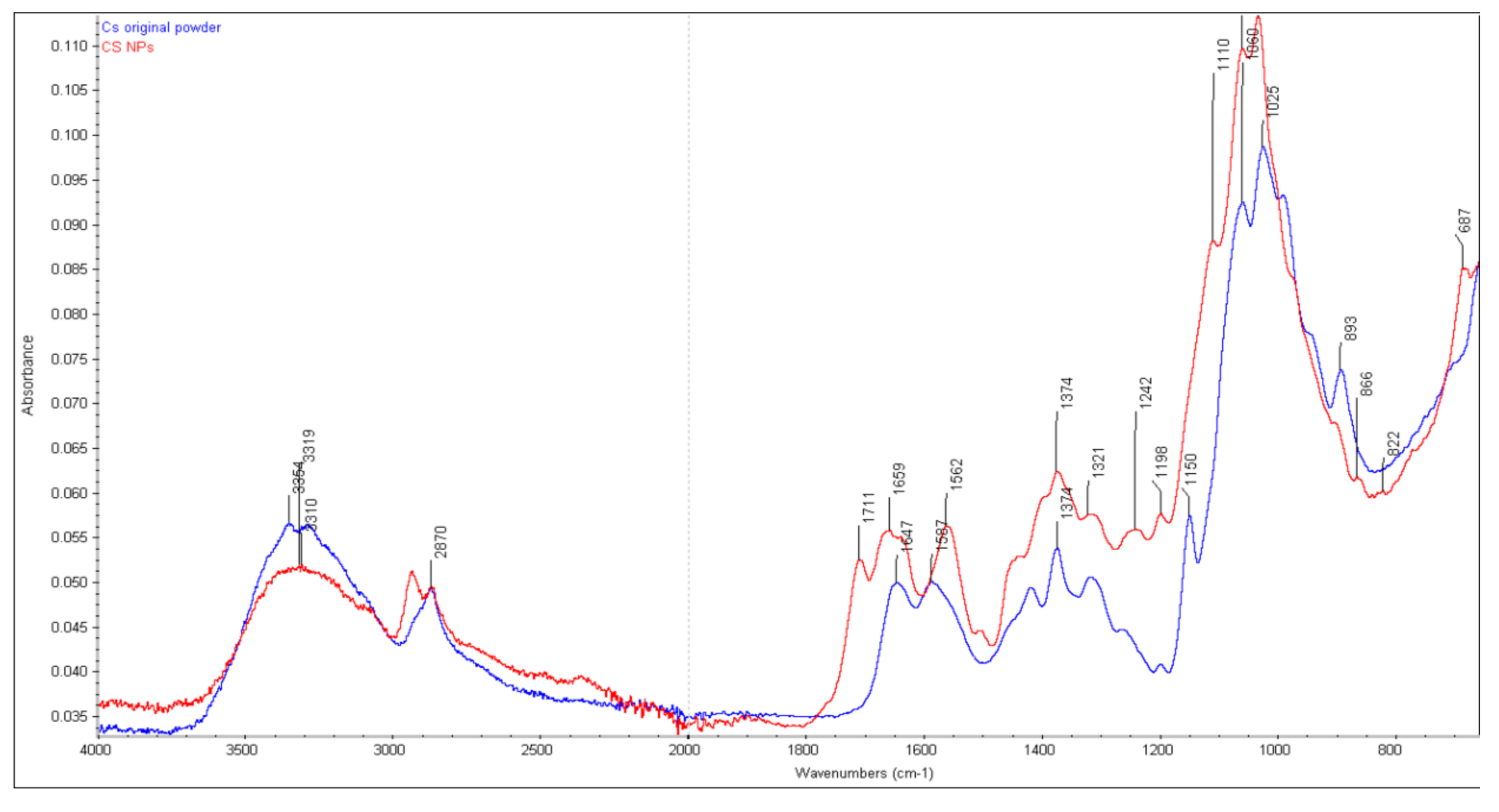
FTIR spectra of CS original powder and CS NP before incubating in 0.2 mg/mL lysozyme solution.

**Figure 2:**
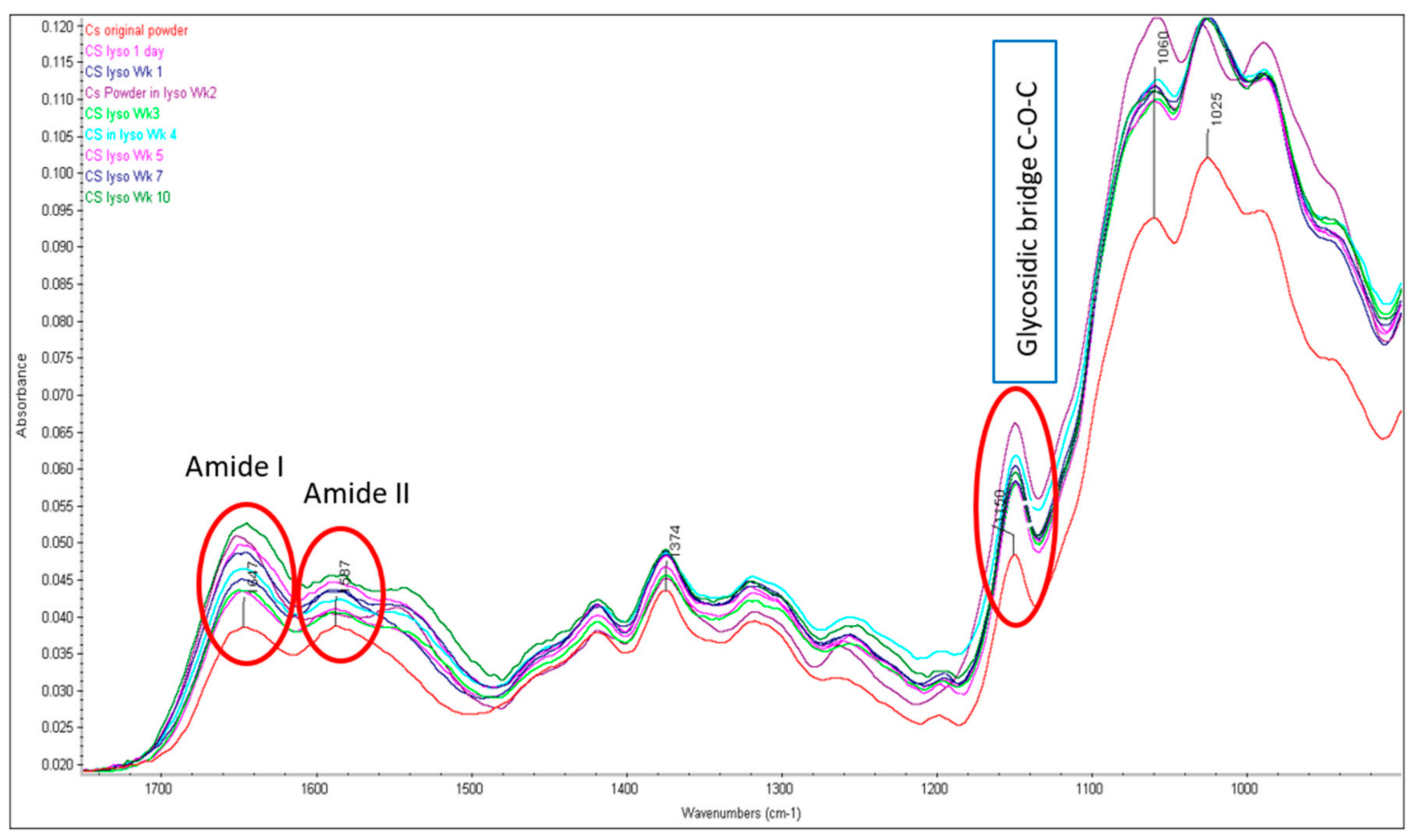
FTIR spectra of CS powders before and after lysozyme treatment (characteristic peaks for amide I, II and glycosidic bonds are highlighted).

FTIR spectra were obtained of the original CS powder and glutaraldehyde crosslinked CS NPs that were incubated in 0.2mg/mL lysozyme solution (pH 7.4) at 37 °C. Several characteristic bands of CS raw material can be identified from FTIR spectra (Figure 1) of the original material. The overlapped peaks of the O-H and N-H stretching were recorded at 3356 cm^−1^. At 2870 cm^−1^, the peak of CH_2_ stretching vibration was recorded, which corresponded to the C-6 of the pyranose ring. The characteristic peaks at 1647 cm^−1^ and 1587 cm^−1^ with high intensity were attributed to the vibration of amide I (C=O stretching) band and amide II (NH bending) band, respectively, of the remaining N-acetyl groups from the parent chitin. The peak at 1150 cm^−1^ is attributed to the asymmetric stretching of the oxygen atom from the C-O-C bridge and carbon atom from the ring (glycosidic bond) and skeletal vibration involving the O-C-O stretching at 1059 cm^−1^ and the polysaccharide structure at 1025 cm^−1^, which are in agreement with others [12,29,30]. A minor shifting of the primary amide band at 1650 cm^−1^ (1647 cm^−1^ for original CS) and a weak band at 1547 cm^−1^ (1587 cm^−1^ for original CS) for secondary amide were observed after two weeks incubation (Figure 2). No changes occurred in Week 5 as the amide I (1650 cm^−1^), amide II (1591 cm^−1^) and C-O-C bridge (glycosidic bond at 1150 cm^−1^) are at the same position compared to that of the original CS powder. The observed characteristic peaks for amide I (1645 cm^−1^), amide II (1583 cm^−1^) and glycosidic bond (1149 cm^−1^) after incubation in lysozyme solution for Wk 10 are also very similar to the original CS, which is an indication that no breakdown of CS structures occurred.

#### 2.1.2. Chitosan NPs

**Figure 3:**
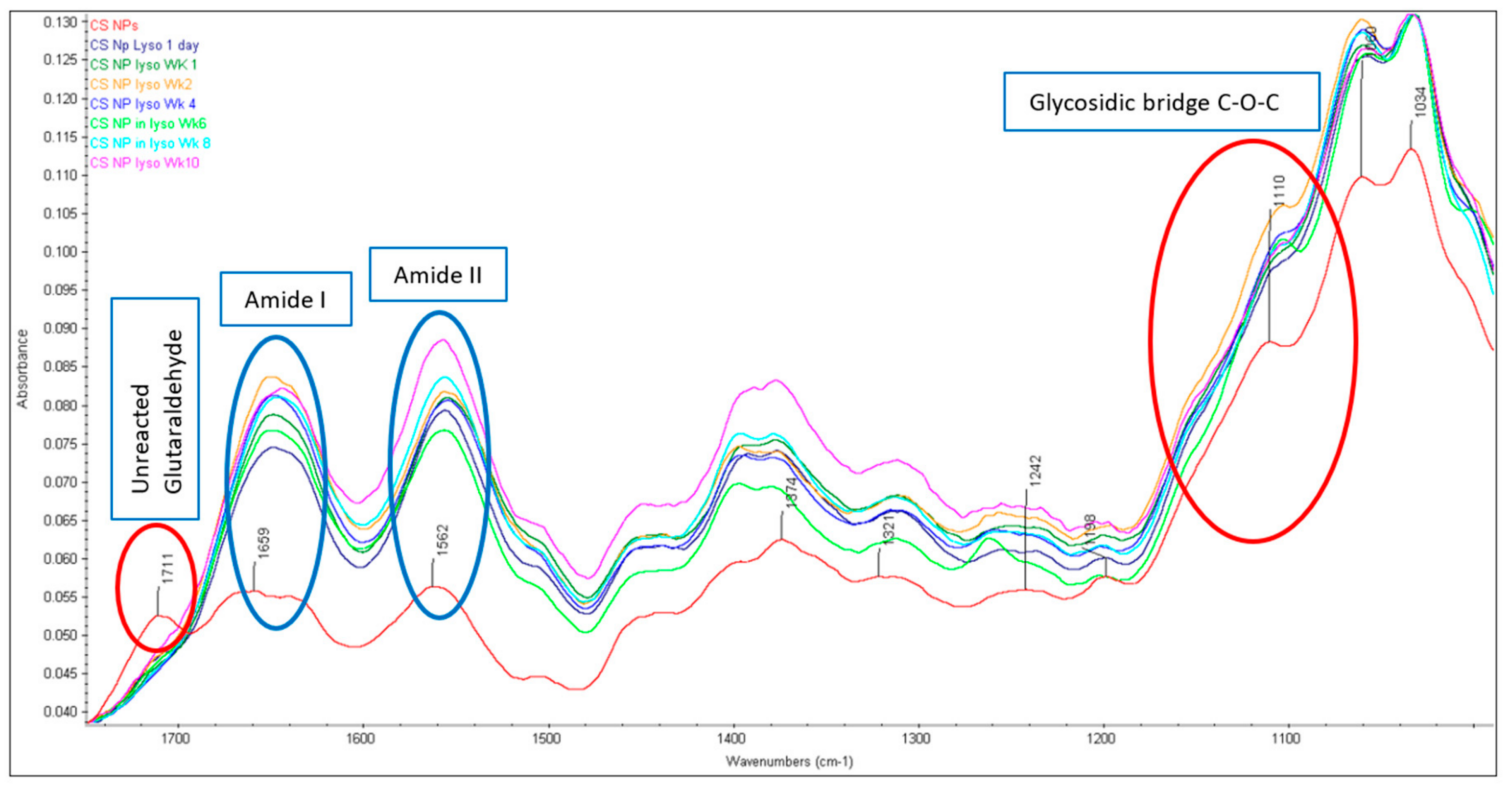
FTIR spectra of CS NPs before and after lysozyme treatment (characteristic peaks for amide I, II and glycosidic bonds are highlighted).

The FTIR spectra of CS NPs formed by crosslinking with glutaraldehyde are presented in Figure 1. The glutaraldehyde crosslinked CS produced three prominent peaks i.e., a sharp peak at 1562 cm^−1^, a wide peak at 1659 cm^−1^ (with a little shoulder peak at 1641 cm^−1^) and another sharp peak 1711 cm^−1^. It is well known that the glutaraldehyde reacts with the amine group of CS and a stable imine (C=N) group (Schiff base) is formed [31]. The amino groups of CS then catalyze the aldol condensation/polymerization reaction favoring the formation of irregular oligomeric products with aldehyde groups that further react with other amino groups available in the nearby CS unit [31,32]. According to the explanation by Liu et al. [32] the peak appearing at 1562 cm^−1^ may be considered as a C=C stretch confirming the aldol condensed oligomer; the very weak peak (little shoulder) at 1641 cm^−1^ is associated with the Schiff base (C=N), the wide peak at higher wavenumber 1659 cm^−1^ is also considered as a C=N stretch; however, in a non-conjugated form; and a low peak at 1711 cm^−1^ is considered as the carbonyl (C=O) band from unreacted aldehyde groups. The specific band stretching of amide I (C=O) band and amide II (NH) at 1647 cm^−1^ and 1587 cm^−1^, respectively, observed in the original CS spectrum, are either absent or convoluted with others (as demonstrated above) in the crosslinked CS nanoparticles [12,33]. Although the nanoparticles were washed with diethyl ether and water to remove excess glutaraldehyde, the absorption band at 1711 cm^−1^ indicated the presence of non-reacted aldehyde group of the crosslinker glutaraldehyde [32]. All of the CS NPs incubated in 0.2 mg/mL lysozyme solution up to week 10 were subjected to the FTIR analysis (Figure 3). The characteristic FTIR peak initially observed at around 1711 cm^−1^ appeared as a weak band after one day and completely disappeared after one week of lysozyme treatment. The actual reason behind this is not clear. Interestingly, no broad peaks initially appearing at 1659 cm^−1^ for the untreated CS NPs were observed after lysozyme treatment; however, two sharp peaks (1554–1558 cm^−1^ and 1644–1656 cm^−1^) were observed in all NPs incubated in 0.2 mg/mL lysozyme solution up to 10 weeks. The characteristic peak at 1150 cm^−1^ associated with the C-O-C bridges observed for original CS, was not apparent at 1150 cm^−1^ for NPs. This peak was suggested to be shifted to 1110 cm^−1^ (prominent peak) and representing the C-O-C bridge in NPs (Figure 1). The peaks around 1110 cm^−1^ (although a little bit weak) for all NPs were seen after lysozyme treatment meaning very insignificant breakdown of the glycosidic bond by lysozyme had occurred at the surface level. The observed sharp peaks between 1060–1032 cm^−1^ represented the O-C-O ring of the CS, was unaffected by the lysozyme during 10 weeks incubation. These peaks are nearly the same as observed in the original CS and the crosslinked NPs.

The primary objective of this study was to investigate the presence or absence of these peaks after incubating in lysozyme solution at pH 7.4. The enzymatic degradation of CS is known to take place via hydrolysis of β-(1→4) N-acetylglucosamine units, which undergo chain scission by the action of lysozymes in the body [34,35,36]. With regards to the glutaraldehyde crosslinked CS NPs, two units of the amine group of CS are crosslinked with one di-aldehyde molecule to form two characteristic imine (N=C) bonds between aldehyde carbons and primary amines based on the Schiff reaction [32]. It was clear that the glycosidic bond (C-O-C bridge at around 1150 cm^−1^) in all samples of original CS appeared at the same position (Figure 2), which indicates that the glycosidic bond had not been hydrolyzed by the lysozyme (0.2 mg/mL) solution. As described above, a wide peak (with a weak shoulder peak at 1641 cm^−1^) at higher wavenumber 1659 cm^−1^ associated with a C=N stretch, was shifted between 1644–1456 cm^−1^ for all NPs incubated in lysozyme solution. The observed sharp peaks between 1644 cm^−1^ to 1656 cm^−1^ (probably the shifted band for N=C stretching) for NPs with high intensity are thought to be convoluted with other bands (Figure 3). To confirm the obvious nature of breaking down the C-O-C bridges by lysozyme, both peak ratios and area ratios of the bands were determined by deconvolution of some characteristic peaks and no correlation was observed. For original CS, it was expected that the C-O-C bridging around 1150 cm^−1^ would decrease relative to a C-H stretch; however, in fact, it increased then decreased with time of lysozyme treatment. In terms of CS NPs, the spectra of C-O-C were very weak or mostly disappeared and thus the correlation analysis could not be performed. However, the presence of weak bands associated with the C-O-C bridges were regarded as the partial breakdown of the glycosidic bond at the surface level.

The FTIR reference spectrum of lysozyme showed three characteristic peaks including amide I (1642 cm^−1^, amide II (1515 cm^−1^), and a very weak signal of amide III (1454 cm^−1^), which is similar to the results demonstrated by others [37]. There could be a possibility that the band stretching of amide I at 1642 cm^−1^ overlapped with the amide bands of original CS (1647 cm^−1^) or CS NPs (amide band at 1659 cm^−1^) incubated in lysozyme solution. It can be noted here that the original CS and CS NPs incubated in lysozyme solution were washed with deionized water in triplicate following centrifugation at 14,000 rpm. Furthermore, these stretching bands in both CS and CS NPs were observed before incubating them in the lysozyme solution. Therefore, there is no possibility that the characteristic amide band of lysozyme was overlapped with the amide bands (1647 cm^−1^) of CS powder and CS NPs (1659 cm^−1^).

### 2.2. SEM Studies

**Figure 4:**
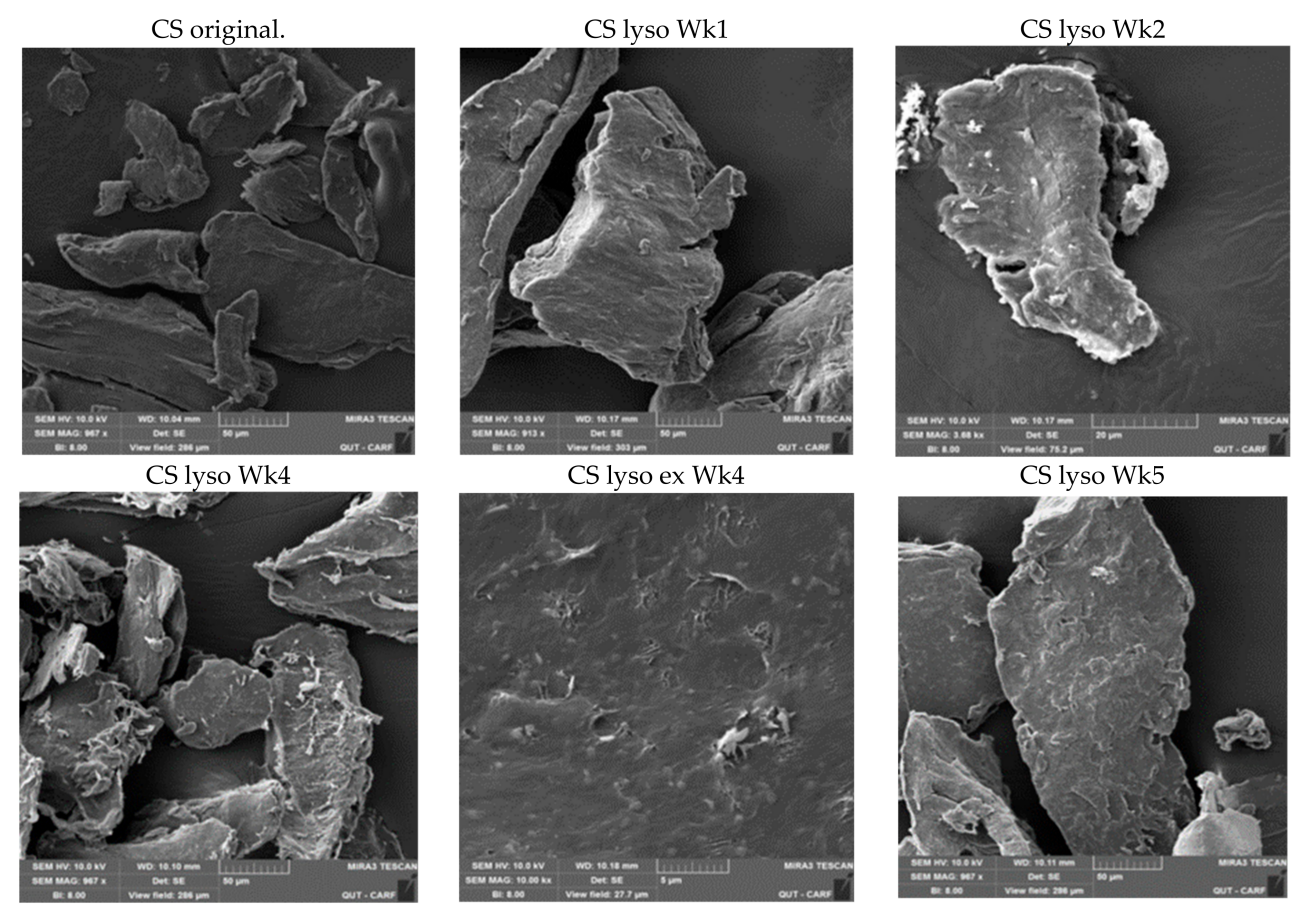
SEM images of CS before and after incubating in 0.2 mg/mL lysozyme solution at different time intervals.

**Figure 5:**
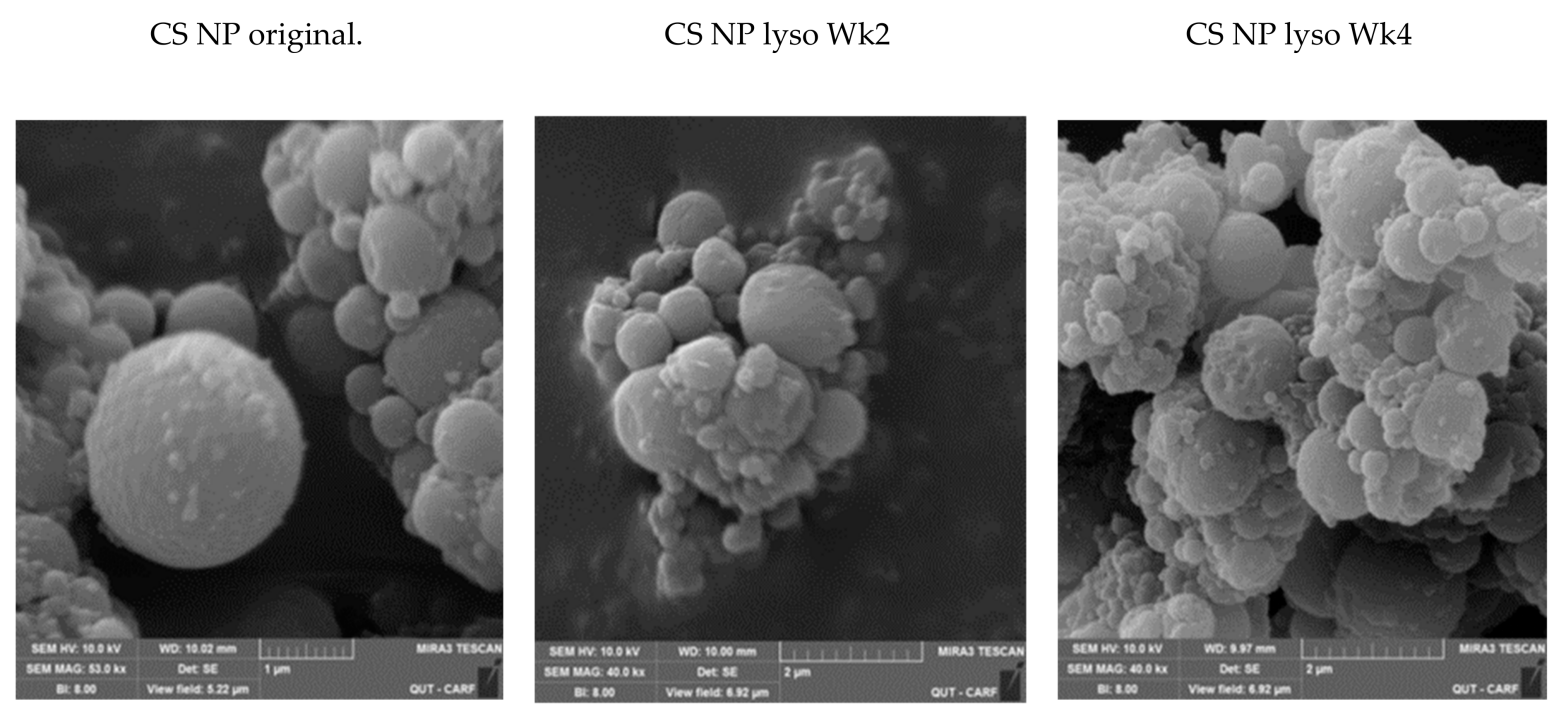

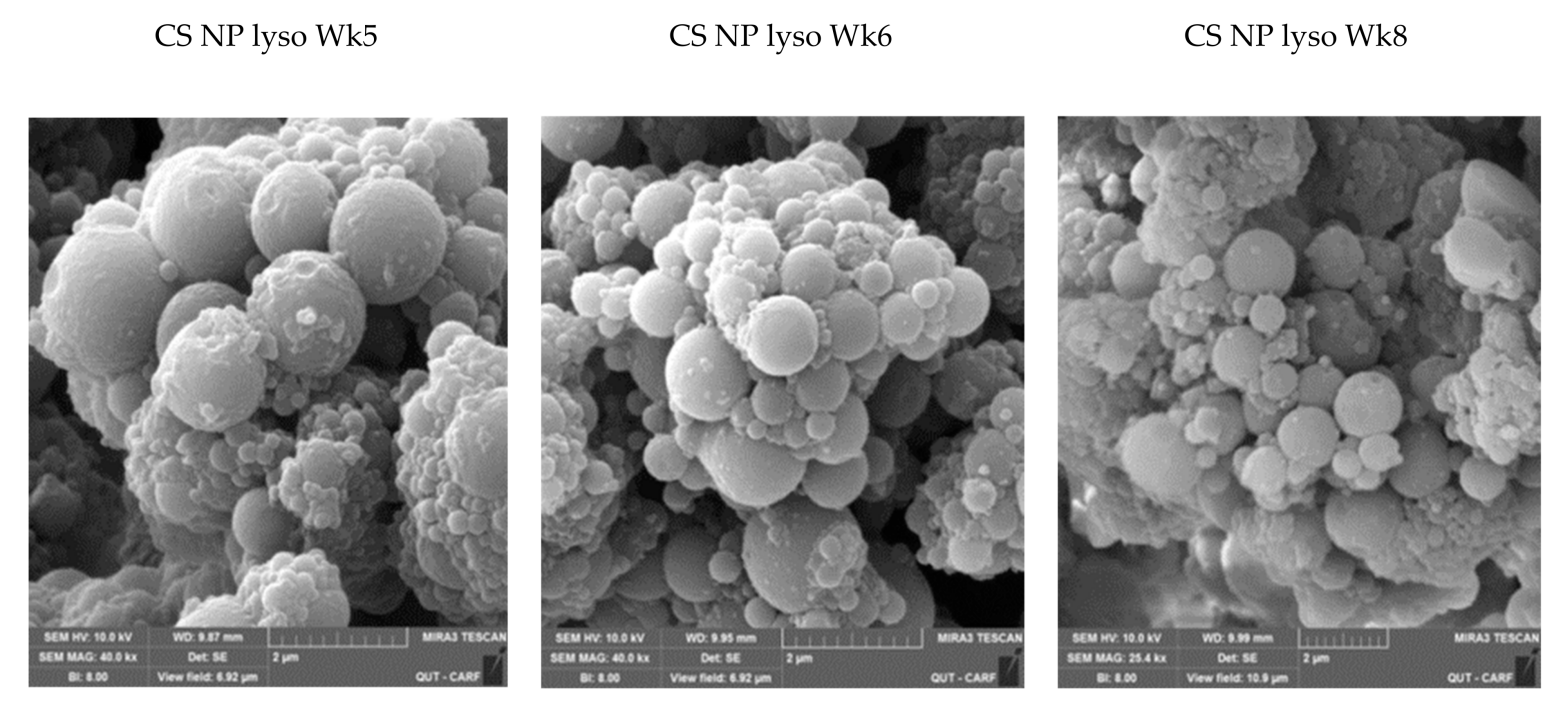
SEM images of CS NPs before and after incubating in 0.2 mg/mL lysozyme solution at different time intervals.

**Figure 6:**
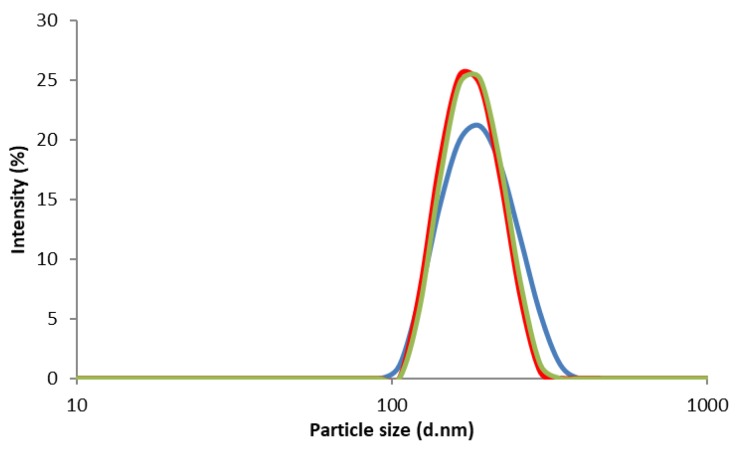
Particle size and size distributions of CS NPs.

**Figure 7:**
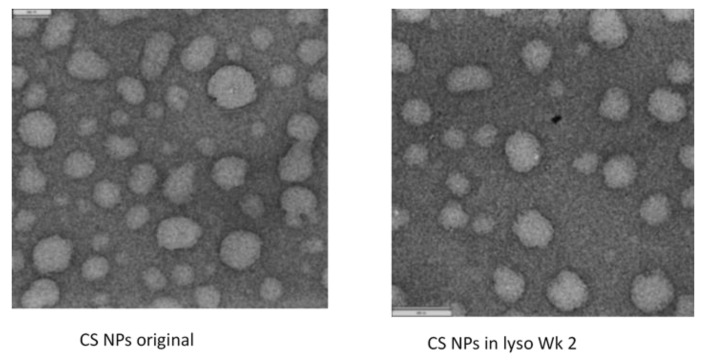
TEM images of CS NPs before and after incubating in 0.2 mg/mL lysozyme solution at 37 °C (TEM images for samples incubated up to 10 weeks could not be performed).

The SEM images of the CS powder and CS NPs before and after incubation in a lysozyme solution at 37 °C are presented in Figure 4 and Figure 5, respectively. The particles look agglomerated and no differences in images (CS powder and CS NPs) before and after incubation up to week 10, were observed. Particle sizes or shapes of the treated samples are similar to those of original images (Figure 4 and Figure 5). This outcome further supports the conclusion that the CS and CS NPs were not degraded by the lysozyme solution. The lysozyme treated CS powder surface (week four) appeared to have some sort of surface digestion; however, the close-up view (Figure 4 CS lyso ex WK4) of the surface showed no signs of surface digestion by lysozyme. It can be noted here that the particles initially (immediately after preparation) showed a unimodal size distribution (Figure 6) with size ranging between 167–190 nm; however, agglomerated after freeze-drying and deagglomerated in lysozyme solution as presented by TEM images (Figure 7). The TEM images are not very clear for detecting the surface digestion by lysozyme; however, the SEM images clearly showed no changes on the particle surface. This outcome suggests no evidence of degradation, which was supported by the FTIR data.

### 2.3. UV-VIS

**Figure 8:**
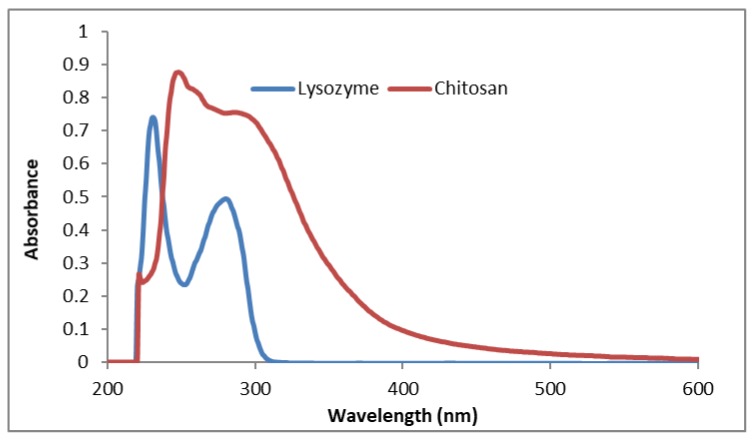
UV-VIS spectra of CS solution (1.0% *w/v*) in acetic acid (1% *v/v*) and lysozyme solution (0.2 mg/mL) in PBS (pH 7.4).

To understand more about the CS breakdown in lysozyme solution, initially, we prepared the original CS solution (1% *w*/*v*) in 1.0% (*v*/*v*) acetic acid (measured pH of 3.97) to find out the characteristic UV-VIS peaks and the results are presented in Figure 8. The absorption spectra of the original CS in acetic acid solution showed a characteristic peak at 247 nm, a very weak band at 259 nm and a broad peak between 290 nm to 300 nm. The broad band around 290 nm may be the characteristic peak for the presence of a set of amino groups [37]. The UV-Vis spectrum of the lysozyme solution was also recorded to determine whether the lysozyme interferes with the absorption peaks of CS. The 0.2 mg/mL lysozyme solution showed two peaks i.e., one at 230 nm (sharp) and the second one at 278 nm, which is similar to results from other investigators [37]. CS solutions are known to absorb in the UV-visible region with a broad maximum at about 290–300 nm. Qasim et al. [36] reported a characteristic absorption band at 200 nm for plain CS and two new absorption bands at 230 and 290 nm which are related to the carboxylic acid and aldehyde groups, respectively, produced due to the oxidative degradation of glycosidic bonds of CS [36]. The authors also emphasized that these two bands vary in intensity over the degradation period; however, no information in terms of the plain chitosan solution, the concentration of lysozyme and pH of the degradation media were provided. Herein, we used lysozyme enzyme solution (0.2% *w*/*v*) in PBS as the degradation media where the breakdown of glycosidic bond can occur by hydrolysis.

**Figure 9:**
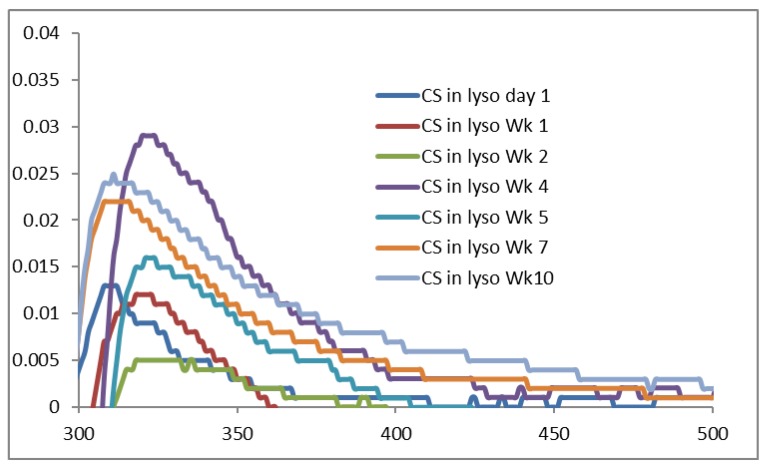
UV-VIS spectra of CS original powder incubated in 0.2% lysozyme solution at 37 °C.

The UV-VIS spectra of original CS powder incubated in lysozyme solution are presented in Figure 9, where very weak absorption peaks are produced between 310 nm to 321 nm. These data indicate that a very insignificant amount of CS is dissolved in the lysozyme solution during the incubation period. However, the FTIR data and the SEM images, although they have different principles and sensitivity, do not support this conclusion. There is the least possibility of CS dissolution at pH above 6.5 (pKa of CS is 6.3 to 6.5) where CS with 92% DD is nearly insoluble and thus the degradation of original CS in lysozyme solution was not observed.

**Figure 10:**
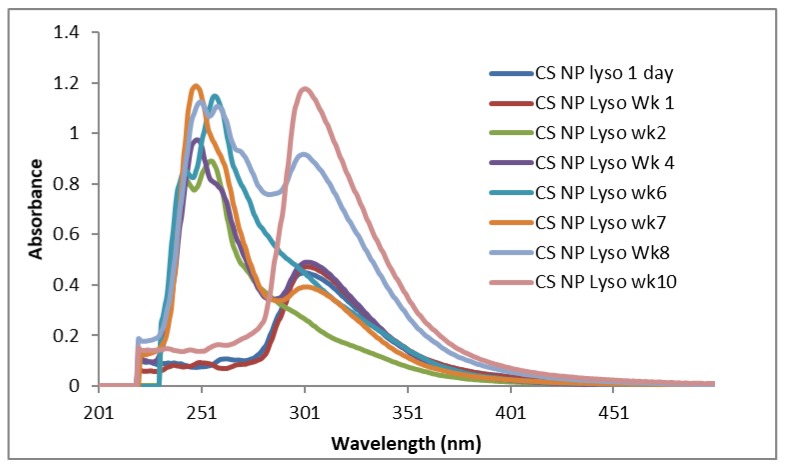
UV- Vis spectra of the supernatant of CS NPs incubated in 0.2% lysozyme solution at 37 °C.

The CS nanoparticles in lysozyme solution incubated at 37 °C showed similar UV-Vis spectra (Figure 10) (except Wk 6 sample, which showed only one sharp peak at 246 nm). Samples incubated for 24 h and seven days showed significant peaks between 298–301 nm, which might be considered as the initial breakdown of CS in a short time. Samples of Wk 4, Wk 7, Wk 8, and Wk 10 showed the right shifting of their first peaks with similar sharp peaks between 298–301nm (Figure 10), which might be considered as a sign of subsequent NPs degradation in lysozyme solution after 3 weeks.

Recently, using the medium molecular weight (MMW) CS, Qasim et al. [36] demonstrated a strong absorption band of CS at 200 nm, which is absent in our study. They also found prominent bands in the range of 200–300 nm which was an indication of hydrolytic scission of β (1→4) glycosidic bonds of CS. Although our study outcomes are different from those reported by Qasim et al. [36], prominent bands for all samples were observed around 245 nm, which might be due to n-σ* (nonbonding to antibonding) transition of amino groups. Another prominent band at 254–256 nm could be due to the typical carbonyl group (π-π* transition) of CS [38]. The absorption bands for CS NPs incubated for 24 h, Wk 1, Wk 4, Wk 7, Wk 8 and Wk 10, are shifted to the right with characteristic sharp bands between 298–309 nm (n-π* transition of carbonyl or carboxyl group), which might be due to the hydrolytic breakage of glycosidic bonds [38]. This indicates that the surface of the CS NPs degraded (breakage of C-O-C bridge) in the first 24 h (weak intensity UV-VIS peak at 298 nm, Figure 10). Similar pattern of UV-VIS spectra for CS NPs observed in Wk 1, and from Wk 4 to Wk 10 (except Wk 6). Although the actual mechanism behind this is unclear; however, it can be presumed that the GlcNAc units linked by glycosidic bonds in the crosslinked CS might not properly expose to lysozyme and the NPs surface glycoside bridges could have interacted with lysozyme that led to the breakdown of the glycosidic bond in 24 h or in Wk 1 (Figure 10). The subsequent breakdown of glycosidic bonds occurred after 3 to 4 weeks (samples of Wk 4- Wk 10, except Wk 6) of lysozyme treatment. The levels of CS acetylation and the distribution of N-acetyl groups along the CS chain have shown to influence its conformation and physicochemical properties ie., solubility, pKa etc [39]. Depending on the DDA, GlcN-GlcNAc disaccharides in CS showed high conformational flexibility, which influenced the spatial distribution of glycosidic bonds in the CS chain and subsequent hydrogen bonding in solution [40]. It was further shown that the CS chains with above 50% deacetylation (DA) are more rigid than CS with lower DA (i.e., CS with high DDA is stiffer), which might influence the C-O-C conformations as well as hydrogen bonding in CS chains. It has been reported that at least 3–4 acetylated units in chitosan is required for lysozyme binding to allow enzymatic cleavage of the glycosidic bond [41]. Therefore, the number of acetylated units in CS chains is also a factor that influences the hydrolysis of the polymer. The CS polymer with 92% DDA used in this study probably doesn’t have sufficient acetylated units for cleavage by lysozyme and that’s why no degradation of CS occurred as evidenced by FTIR (Figure 2) and UV-VIS data (Figure 9). In case of CS NPs, some GlcNAc being exposed on the surface and thus accessible to the enzyme for interaction via hydrogen bonding and led to the breakdown of the glycosidic bond as evidenced by both FTIR (Figure 3) and UV-VIS data (Figure 10). Although SEM images showed no such changes on the NPs surface, the FTIR showed no peaks around 1150 cm^−1^ (the characteristic band stretching for C-O-C bond); however, very weak peaks were observed around 1110 cm^−1^, which indicated the partial breakdown of C-O-C bridges on NPs surface occurred due to lysozyme treatment. UV-VIS spectra of NPs from Wk4 to Wk10 showed a similar pattern as NPs might take nearly 3 weeks to be swelled up that presumably allowed more exposure of GlcNAc on the surface for lysozyme to act onto the opened C-O-C bridges, which was broken in subsequent weeks (Wk 4–Wk 10, except Wk 6). With regards to the original CS, it could be expected that the CS with 92% DDA has fewer glucosamine to N-acetylglucosamine linkages resulting in less flexible CS chains [40] without favorable hydrogen bonding with lysozyme. This result in limited interactions with lysozyme and no breakdown of C-O-C bonds observed, which was confirmed by the presence of the characteristic FTIR bands at 1150 cm^−1^.

### 2.4. Impact of pH

With regards to the impact of pH on CS degradation, a trend of decreasing pH of the lysozyme solutions containing CS and CS NPs with increasing incubation time (Table 1) was observed. Interestingly, the pH of the CS solutions, but not the CS NP solutions, showed an unexpected change and started to increase at week 8. The pKa of chitosan is 6.5 and thus CS is expected to be partially dissolved around the pH similar to pKa. The CS is soluble in acidic solution at low pH (<6) and therefore, it was presumed that an insignificant amount of the CS was dissolved or degraded from NPs after Wk 4 and produced sharp peaks between 298–301 nm, which was an indication of the breakdown of glycosidic bond [38]. The FTIR (although operated by different principles with different sensitivity) data did support the disappearance of the glycosidic bonds, which confirmed an indication of bond breakage in lysozyme solution. Recently, Peng et al. [42], studied the degradation of 5-Fluorouracil (5-FU) loaded CS microspheres in lysozyme solution, and increased pH was observed with increased incubation time until 20 days, which is opposite to our study. The reason behind increased pH was probably due to the released 5-FU in the lysozyme solution which caused to increase the pH of the solution.

Depending on the MW, DD of CS, and pH of the medium, the degradability studies of CS or its NPs by lysozyme is not straightforward. Grenha et al. [28] demonstrated the reduction of particle size of TPP crosslinked CS NPs (degree of deacetylation 86%) in 0.2 mg/mL lysozyme solution. Similar outcomes were also demonstrated by Poth et al. [27], who used CS with DD of less than 50%. In another study, CS with a DA <17% was found to be less susceptible to enzymatic degradation [14]. The authors explained that the degradation was pH dependent and CS was insoluble at a pH above 6.5. As CS is practically insoluble at a pH above 6.5, the degradation of CS by lysozyme did not occur due to limited accessibility of the binding sites for the enzyme with the glycosidic bond. Hou et al. [43] investigated the degradation of TPP crosslinked CS NPs by determining the rate of NPs mass loss in 100 µg/mL (0.1 mg/mL) lysozyme solution, which mimics the in vivo physiological conditions as the lysozyme concentration in serum is between 0.95–2.45 µg/mL [44]. The degradation of TPP-CS NPs measured as the mass loss, was dependent on the concentration of TPP and a higher crosslinking density caused a reduction of the penetration and accessibility of the lysozyme to the particle network and subsequently slowed down the degradation of CS NPs. Very recently, Mazancova et al. [45] showed that the TPP crosslinked CS complexes dissociate into separate components at higher pH and complete dissociation into free CS and TPP occurred at a pH of physiological range, where CS is practically insoluble; however, they did not study the degradation of dissociated CS or TPP crosslinked NPs.

Tomihata and Yoshito [46] reported that CS could be hydrolyzed by a high concentration of lysozyme solution (4 mg/mL) at the low degree of deacetylation (DD) and significant degradation of CS at a DD value of higher than 73% was observed. CS with DD above 73% was reported to be virtually resistant to a lysozyme solution of 4.0 mg/mL [46,47]. A negligible degradation rate of highly deacetylated (fraction of acetylation 0.04) chitosan by lysozyme was observed [35]. It has also been reported that lysozyme is ineffective in catalyzing the hydrolysis of CS with deacetylation degree higher than 95% [48]. In our study, the degradability of CS (DD 92%) and glutaraldehyde crosslinked CS NPs was investigated in 0.2 mg/mL concentration of lysozyme solution similar to biological lung environment and some degree of degradation in this lysozyme solution was expected. The data generated by both FTIR and UV-VIS suggested an insignificant breakdown of C-O-C bonds occurred in CS NPs (very weak bands around 1110 cm^−1^ associated with the glycosidic bonds). No breakdown of this bond in the original CS was observed. Therefore, it can be noted here that CS with high DD is not degradable in the lung environment and is unsuitable for pulmonary delivery of drugs; however, further studies are warranted to ensure the complete degradation before application to pulmonary drug delivery. More studies are required to understand the degradation of CS with different DD, and the impact of the concentration of CS and glutaraldehyde in CS NPs on the particle formation and degradation in the lung environment.

## 3. Materials and Methods

### 3.1. Materials

Chitosan: Low molecular weight chitosan (MW 50–190 kDa; degree of deacetylation DDA 92%), PBS and lysozyme were purchased from Sigma-Aldrich Pty Ltd (Castle Hill, NSW, Australia). 50% aqueous glutaraldehyde (MW 100.1 g/mol, density: 1.06 g/cm³) was from Thermofisher (Lancashire, UK). Span 80 was obtained from PCCA (Matraville, NSW, Australia). All other chemicals/ reagents used were analytical grade.

### 3.2. Methods

#### 3.2.1. Preparation of Glutaraldehyde Crosslinked CS NPs

The nanoparticles were prepared using the method of Wang et al. [12]. Briefly, the blank CS particles were prepared by dissolving CS powder in 2% *v*/*v* acetic acid solution at pH 3.5. Paraffin oil containing span 80 was added drop-wise and the mixture homogenized to obtain the W/O emulsion. The particles were formed by adding 50% glutaraldehyde solution, washed with hexane and diethyl ether, centrifuged and freeze-dried at −80 °C.

#### 3.2.2. Particle Size and Size Distribution of CS NPs

The average diameter of the prepared CS NPs was measured by dynamic light scattering (DLS) with Zeta-Sizer Nano ZS (Malvern Panalytical Ltd Malvern, UK). CS particles were suspended in phosphate buffered saline (PBS, pH 7.4) and sonicated for 5 min. Refractive index of CS used was 1.523. All runs were performed at 25 °C in triplicate.

#### 3.2.3. Degradation Studies

A phosphate buffered saline (PBS) tablet (Sigma Aldrich) was dissolved in 200 mL milliQ water and the pH was measured immediately after preparation. Using the prepared PBS solution, a 0.2 mg/mL lysozyme (pH 7.4) solution was produced as this concentration of lysozyme is the maximum level available in human tracheobronchial secretions) [28]. Suspensions of CS powder and CS NPs in the prepared PBS solution (1.0 mg/mL) were prepared and sonicated for 5 min. All samples were clamped with an orbital shaker at 50 rpm and incubated at 37 °C. The pH of the suspensions was measured weekly and fresh lysozyme solution was added once per week [43]. The suspensions were centrifuged at 14,000 rpm for 4 min, the supernatant was removed and the pellet was washed with deionized water (×3). The samples were freeze-dried at −80 °C for 24 h and subjected to ATR-FTIR and SEM scanning.

#### 3.2.4. Attenuated Total Reflectance-Fourier Transform Infrared (ATR-FTIR)

The ATR-FTIR spectra of all samples were obtained using a Thermo Nicolet Nexus 870 FTIR spectrometer (Triad Scientific Inc, NJ, USA) equipped with a single reflection diamond crystal attenuated total reflectance (ATR) accessory with an angle of incidence 40° and a deuterated triglycine sulfate (DTGS). A small amount of sample was placed on the top of the diamond crystal and secured with a high-pressure clamp. All spectra were collected at a resolution of 8 cm^−1^ and 64 scans in the range of 4000–500 cm^−1^ and analyzed using the spectral analysis software OMNIC (Nicolet Instrument Corp., Version 7.2, Madison, WI, USA). To evaluate the chemical structure of CS, the band relating to the C-O-C bridge, which is located at 1150 cm^−1^ (anti-symmetric stretching of C-O-C bridge) was examined as evidence of the presence of glycosidic bonds before and after lysozyme treatment.

#### 3.2.5. Transmission Electron Microscopy (TEM)

The TEM images of the incubated NPs were taken with a JEOL JEM -1400 TEM (JEOL Tokyo, Japan) every two weeks. A small amount (10 µL) of the suspension was dropped onto a copper grid, and excess water was absorbed by a piece of filter paper. A drop of 2% uranyl acetate negative stain was added before drying at room temperature. The examinations were carried out at the accelerating voltage of 200 kV without any further modification or coating.

#### 3.2.6. Scanning Electron Microscopy (SEM)

The original CS, lysozyme treated CS and lysozyme treated CS NPs were examined by SEM (MIRA 3, TESCAN, Brno, Czech Republic).

A small amount of dried powder was sprinkled onto a silicon wafer adhered to an aluminum stub through a carbon adhesive tape. The air-dried specimen stubs were coated with a conductive layer of sputtered platinum (argon gas pressure of 0.5 mbar, current of 30 mA, and a coating time of 75 s), followed by observing secondary electron images under a high vacuum with an accelerating voltage of 5 kV and a working distance of 6.8 mm. A variety of photomicrographs with different magnifications were captured for all samples at different time intervals.

#### 3.2.7. pH Analysis

Using a pH meter (Mettler Toledo AG, Zurich, Switzerland), the pH measurements of the supernatant were carried out at each time by first calibrating the pH meter in standard solution (pH 7) and then immersing the probe into the solution/suspensions until the pH reading was stabilized. CS powder and NPs suspensions in 0.2 mg/mL lysozyme solution were the test samples and lysozyme (in PBS solution) was considered as a reference standard.

#### 3.2.8. UV-VIS Spectroscopy

Using a UV-VIS spectrophotometer (DeNovix Inc DS 11+, Wilmington, DE, USA) the spectrophotometric analysis of the prepared CS solution (1% *w*/*v*) in acetic acid (1% *v*/*v*) and the supernatant of the degradation media, was performed. The CS solution was centrifuged for 5 min at 16,000 rpm and analysis was carried out at absorbance values between 190–500 nm. A 0.2 mg/mL lysozyme solution in PBS was used as the reference.

## 4. Conclusions

The degradability of CS and NPs in lysozyme is very complicated as the DD and MW are important factors. In our study, a 0.2 mg/mL concentration of lysozyme could not degrade the LMW original CS with a DD of 92%. Both the FTIR and UV-VIS data demonstrated that no C-O-C bridge breakdown of original CS occurred in lysozyme solution during the incubation period. Interestingly, very weak FTIR bands associated with the C-O-C bridges were observed for CS NPs incubated in lysozyme solution. The UV-VIS spectra of the CS NPs also showed the signs of glycosidic bond breakage. The outcome of this study indicated that the original CS with 92% DD was not degradable; however, its NPs were partially degraded after 10 weeks incubation in 0.2 mg/mL lysozyme solution. No signs of breaking the covalent bonds between the crosslinker glutaraldehyde and CS were observed as determined by the FTIR spectra. The lung is a body part from where no other alternative routes are available to eliminate the materials after inhalation. The outcomes of this study revealed that CS with 92% DD and its NPs are not suitable for lung drug delivery. Therefore, a deeper knowledge of the in vitro/in vivo degradation of CS with varying degrees of DD and their subsequent toxicity of CS and its NPs for pulmonary drug delivery is warranted.

## Figures and Tables

**Table 1:** Measured pH of the lysozyme solution containing different samples at different time intervals.

Samples	Wk1	Wk2	Wk3	Wk4	Wk5	Wk6	Wk7	Wk8	Wk9	Wk10
CS in Lyso	7.45	7.24	7.25	7.25	7.08	6.66	6.60	6.93	7.04	7.18
CS NP in Lyso	7.23	7.24	7.27	7.02	6.99	6.70	6.69	6.88	6.66	6.28
